# Treatment outcomes and their determinants in HIV patients on Anti-retroviral Treatment Program in selected health facilities of Kembata and Hadiya zones, Southern Nations, Nationalities and Peoples Region, Ethiopia

**DOI:** 10.1186/s12889-015-2176-5

**Published:** 2015-08-27

**Authors:** Wondimu Ayele, Afework Mulugeta, Alem Desta, Felicia A. Rabito

**Affiliations:** School of Public Health, Addis Ababa University, P.O. Box 9086, Addis Ababa, Ethiopia; Department of Public Health, Mekelle University, Mekelle, Ethiopia; Department of Public Health, Tulane University, New Orleans, LA USA

## Abstract

**Background:**

Ethiopia has been providing free Antiretroviral Treatment (ART) since 2005 for HIV/AIDS patients. ART improves survival time and quality of life of HIV patients but ART treatment outcomes might be affected by several factors. However, factors affecting treatment outcomes are poorly understood in Ethiopia. Hence, this study assesses treatment outcomes and its determinants for HIV patients on ART in selected health facilities of Kembata and Hadiya zones.

**Methods:**

A retrospective cohort study was conducted on 730 adult HIV/AIDS patients who enrolled antiretroviral therapy from 2007 to 2011 in four selected health facilities of Kembata and Hadiya zones of Southern Ethiopia. Study subjects were sampled from the health facilities based on population proportion to size. Data was abstracted using data extraction format from medical records. Kaplan-Meier survival function was used to estimate survival probability. Cox proportional hazards regression model was used to identify factors associated with time to death.

**Result:**

Median age of patients was 32.4 years with Inter Quartile Range (IQR) [15, 65]. The female to male ratio of the study participants’ was 1.4:1. Median CD4 count significantly increased during the last four consecutive years of follow up. A total of 92 (12.6 %) patients died, 106(14.5 %) were lost to follow-up, and 109(15 %) were transferred out. Sixty three (68 %) deaths occurred in the first 6 months of treatment. The median survival time was 25 months with IQR [9, 43]. After adjustment for confounders, WHO clinical stage IV [HR 2.42; 95 % CI, 1.19, 5.86], baseline CD4 lymphocyte counts of 201 cell/mm^3^ and 350 cell/mm^3^ [HR 0.20; 95 % CI; 0.09−0.43], poor regimen adherence [HR 2.70 95 % CI: 1.4096, 5.20], baseline hemoglobin level of 10gm/dl and above [HR 0.23; 95 % CI: 0.14, 0.37] and baseline functional status of bedridden [HR 3.40; 95 % CI: 1.61, 7.21] were associated with five year survival of HIV patients on ART.

**Conclusion:**

All people living with HIV/AIDS should initiate ART as early as possible. Initiation of ART at the early stages of the disease, before deterioration of the functional status of the patients and before the reduction of CD4 counts and hemoglobin levels with an intensified health education on adherence to ART regimen is recommended.

## Background

An estimated 34.0 million adults worldwide were living with Human Immunodeficiency Virus (HIV) and of these 2.5 million were newly infected with the virus and 1.7 million died of HIV/ Acquired Immune Deficiency Syndrome (AIDS) in 2011 [1]. In Sub–Saharan Africa, more than 23.5 million people were living with HIV, 1.8 million become newly infected and 1.3 million died of (AIDS) at the end of 2011 [1].

HIV management is currently in an era of effective ART. In the last 29 years, modern drug discovery and have development has transformed HIV-1 disease into a treatable chronic infectious disease [2]. In 2009 alone, 1.2 million people received ART for the first time worldwide, an increase in the number of people receiving treatment by 30 % in a single year. Overall, the number of people receiving therapy has grown 13-fold, more than five million people in low and middle-income countries, since 2004 [3].

Since 2005, the Ethiopian government in collaboration with its partners has been providing free antiretroviral drugs for eligible HIV/AIDS patients in order to reduce epidemics and improve the quality of life for those living with the virus. Of the more than 1.2 million individuals living with the virus, 397,818 were eligible in 2010/11 for antiretroviral treatment [4]. There were about 743 health facilities that provide ART for 333,434 HIV/AIDS patients in 2010/11 [4].

According to the 2011 Ethiopian Demographic and Health Survey, the national prevalence of adult HIV was 2.4 and 1.0 % in SNNPR [5]. Regarding the distribution, urban areas had much higher prevalence (4.0 %) than rural areas (0.6 %). Moreover, the prevalence was higher among females (1.9 %) than males 1.0 % [5].

The use of antiretroviral medicines has dramatically reduced AIDS related illnesses and death in countries where these drugs are widely accessible [6].

HIV incidence has fallen by more than 25 % between 2001 and 2009 in 33 countries. Of these countries, 22 are from sub-Saharan Africa [3]. Early reports from ART programs in resource-limited settings have been promising, with virological efficacy comparable to industrialized countries [7]. Nevertheless, mortality has been high, particularly during the first months after initiating ART [7, 8]. While evidences have proven that ART improves the survival time and quality of life of HIV patients, several clinical and socio demographic factors contributing to this high mortality and poor treatment outcome are not well understood in Ethiopia. Hence, this study was designed to investigate the treatment outcomes and its determinants for HIV patients on ART in selected health facilities of Kembata and Hadiya zones, Southern Nations, Nationalities and Peoples Region, Ethiopia.

## Methods

### Study area and period

The study was conducted in four selected health facilities from Kembata and Hadiya zones, South Nations, Nationalities and Peoples Region [SNNPR], Ethiopia. SNNPR is one of the largest regions in Ethiopia, accounting for more than 10 percent of the country’s land area and the current population is approximately 17 million with an average household size of 4.8 in 2007. More than 91 percent of the SNNPR population lives in rural areas [9]. The mid-2010 population was estimated at nearly 17,745,000 [10]. The region is divided into 13 administrative zones. But, this study was conducted in two Health Centers and two Zonal Hospitals of Kembata and Hadiya zones. Kembata Zone has ten districts and an estimated total population of 1.2 million. It has one zonal hospital, 30 functional public health centers and 262 health posts among which nine health centers and one zonal hospital are providing ART services for 986 ART patients [11]. Similarly, Hadiya Zone has 11 districts with an estimated total population of 1.5 million. It has one zonal hospital, 37 functional public health centers and 282 health posts among which ten health centers and one zonal hospital are providing ART service for 2367 ART patients. The health service coverage in the two zones is 100 % [12]. The study was conducted from January to February, 2012.

### Study design

A retrospective cohort study was conducted on people living with HIV/AIDS enrolled for ART services in two Health Centers and two Zonal Hospitals of Kambata and Hadiya zones from 2007 to 2011.

### Target and study population

The target population was all adult HIV positive patients enrolled for ART services in SNNPR and the study population was all adult HIV positive patients enrolled on ART from December 2007- December 2011 in four selected health facilities of Kembata and Hadiya zones of SNNP Region. All HIV/AIDS patients who were 15 years old and above and enrolled on ART program for at least two clinical visits in the selected health facilities were included in the study. Patients who started treatment in other places and with incomplete baseline CD4 counts and basic personal information such as hemoglobin level and age were excluded.

### Sample and sampling technique

Purposive sampling was used to include the two zones from the 13 zones of SNNPR. Currently there are 20 health facilities providing ART services in the study area. Among these, six health facilities (two hospital and four health centers) were eligible for this study. Eligible health facilities were stratified into hospital and health centers in each study zone and then one hospital was included from each zone. Since each zone had only one hospital, the two hospitals namely Durame Hospital from Kembata and Hossana Hospital from Hadiya were included as study sites. Among the two eligible health centers from each zone, the health center with the highest number of HIV patients (case load) on ART was selected. Thus, one hospital and one health center were selected from each zone to be included in the study. Finally all eligible patients (730) on ART from the four health facilities were included as study subjects.

### Standard and operational definition

The main outcome variables were death and time to death in any of the five years follow up period. Secondary outcome variable (undesirable outcome) was time when patient was lost to follow up, stopped or dropped out any of the five years follow up period. Survival time was defined as the length of time between ART initiation and death/censure. The predictor (covariate) variables included in the model were socio demographic factors (age, sex, marital status, level of education, religion, residence, employment status, spouse health and ART enrollment condition), past and new opportunistic illness, WHO clinical stage, baseline CD4 cells count, baseline weight, hemoglobin, drug regimen, functional status, adherence to HIV medication, attendance of HIV related training, HIV prophylaxis, type of facility (health center, hospital), tobacco smoking, alcohol drinking and use of other substances.

### Data collection tools and procedures

A structured data extraction format was adopted from ART intake and follow up forms of ART clinics. Six nurses were recruited and one for each health center and two for each hospital were assigned as data collators. Data was collected retrospectively from all eligible medical records of adult HIV/AIDS patients on ART from ART in take form and ART follow up forms.

### Data management and statistical analysis

Data was entered, cleaned and coded and checked for missing values, outliers and inconsistencies using SPSS Version 16. To ensure data quality, data collectors were trained, pre-test was carried out at a health center with similar setting to the study health facilities, filled-in forms were checked for completeness and accuracy and corrected on daily basis before leaving the health facilities and 10 % of data were re-entered and discrepancies reviewed by an independent person. Data analysis was carried out with STATA 11.

Descriptive statistical methods were used to summarize the socio-demographic and clinical characteristics of the study participants. The survival time was calculated in months using the time interval between date of ART initiation and 1) date of event (death) for events, 2) date of transfer for transferred out (TO), 3) first date of the first missed appointment for lost cases and 4) the date in which patient completed the 60 months (five years) follow up. For survival analysis two scenarios were considered separately, a real-case assumption (confirmed dead cases were used as events) and a worst-case assumption (lost cases were also considered as events). Descriptive survival statistical analysis (Kaplan-Meier) was used to estimate survival probability after ART initiation. Cox proportional hazards models were used to examine baseline factors associated with time to death. To evaluate the effects of covariate on patient survival, the Wald test was used to define the variables to be entered into the Cox proportional hazards model. Factors that were associated with time to death at 25 % significant level in the bivariate analysis were included in the final Multivariate analysis. Then important predictor variables were identified by fitting the stepwise Cox’s proportional hazard model to the data set. The results of the final model were expressed in terms of Hazard Ratio (HR) and 95 % confidence intervals (CI) and statistical significance was declared if the p-value is less than 0.05. The presence of potential confounding factors or interaction were diagnosed and treated accordingly. Finally Cox proportional hazards model goodness of fit were assessed by using Cox-Snell residual and Harrell’s Concordance statistic test and the model was fitted accordingly to predicted response variable (Fig. 5).

Ethical clearance was obtained from the Institutional Review Board of the College of Health Sciences of Mekelle University. No information obtained from the medical records was disclosed to any third person. Patient identification variables such as name were not used in the study. This study did not inflict harm on or expose HIV/AIDS patients to unnecessary risk as a result of reviewing their medical records.

## Result

Out of, 730 patients included in the analysis, 270(37 %) were initiated on ART in 2007, While 134(18 %), 160(22 %) 81(11 %) and 85(12 %) were initiated in 2008, 2009, 2010 and 2011 respectively. The study findings revealed that 429 (59 %) study subjects were females. The median age of the patients was 30 with IQR [26, 37] years. Of the study subjects, two-third of them were had no formal education or completed primary education at the time of initiation of their ART treatment. Almost 62 % of the patients were unemployed at the time of initiation of ART and 51.4 % of the patients were enrolled from urban districts. From a total of 730 patients, 178(24.4 %) drink alcohol, 98 (13.4 %) use other substances such as Khat and Shisha and 94 (12.9 %) smoke tobacco at initiation of ARV treatment (Table 1). From 730 patients, 481(65.9 %) had opportunistic infections at the time of treatment initiation and 196(26 %) patients developed opportunistic infections after initiation of ARV treatment.

**Table 1:** Description of demographic and behavioral characteristics of patients at baseline in Durame and Hosanna hospitals and Shinshicho and Hossana health centers, 2007–2011, (*n* = 730)

	Variable	Number	Percent
Sex	Male	301	41.2 %
	Female	429	58.8 %
Age at start of ART by years	15_24	92	12.6 %
	25_34	359	49.2 %
	35__44	197	27.0 %
	45__54	58	7.9 %
	>54+	24	3.3 %
ART initiated by Years	2007	270	37.0 %
	2008	134	18.0 %
	2009	160	22.0 %
	2010	81	11.0 %
	2011	85	12.0 %
Marital status	Single	94	12.9 %
	Married	415	56.9 %
	Separated	51	7.0 %
	Divorced	58	8.0 %
	Widowed	111	15.2 %
	Unknown	1	0.001 %
Level of education	Without formal education	161	22.1 %
	Primary	315	43.2 %
	Secondary	204	27.9 %
	Tertiary	50	6.8 %
Religion	Orthodox	176	24.1 %
	Muslim	91	12.5 %
	Protestant	445	61.0 %
	Catholic	18	2.5 %
Residence	Urban	374	51.4 %
	Rural	354	48.6 %
	Unknown	2	0.003 %
Occupation	Employed	278	38.1 %
	Unemployed	451	61.9 %
	Unknown	1	0.001 %
Condition of the husband/wife	Healthy	127	19.8 %
	Chronically ill	155	24.1 %
	Dead	148	23.0 %
	Unknown	213	33.1 %
Spouse treatment status	Enrolled ART	179	27.8 %
	Not Enrolled ART	254	39.4 %
	Unknown	212	32.9 %
Risk behavior at baseline	Drink alcohol	178	24.4 %
	Smoke tobacco	94	12.9 %
	Use other substances	98	13.4 %
	No use of any substance	360	49.3 %
Attending HIV related training	Yes	358	49 %
	No	372	51 %
Understanding HIV prophylaxis	Good	356	46 %
	Poor	374	54 %
Understanding HIV medication adherence	Good	311	42 %
	Poor	419	58 %

**Fig. 1:**
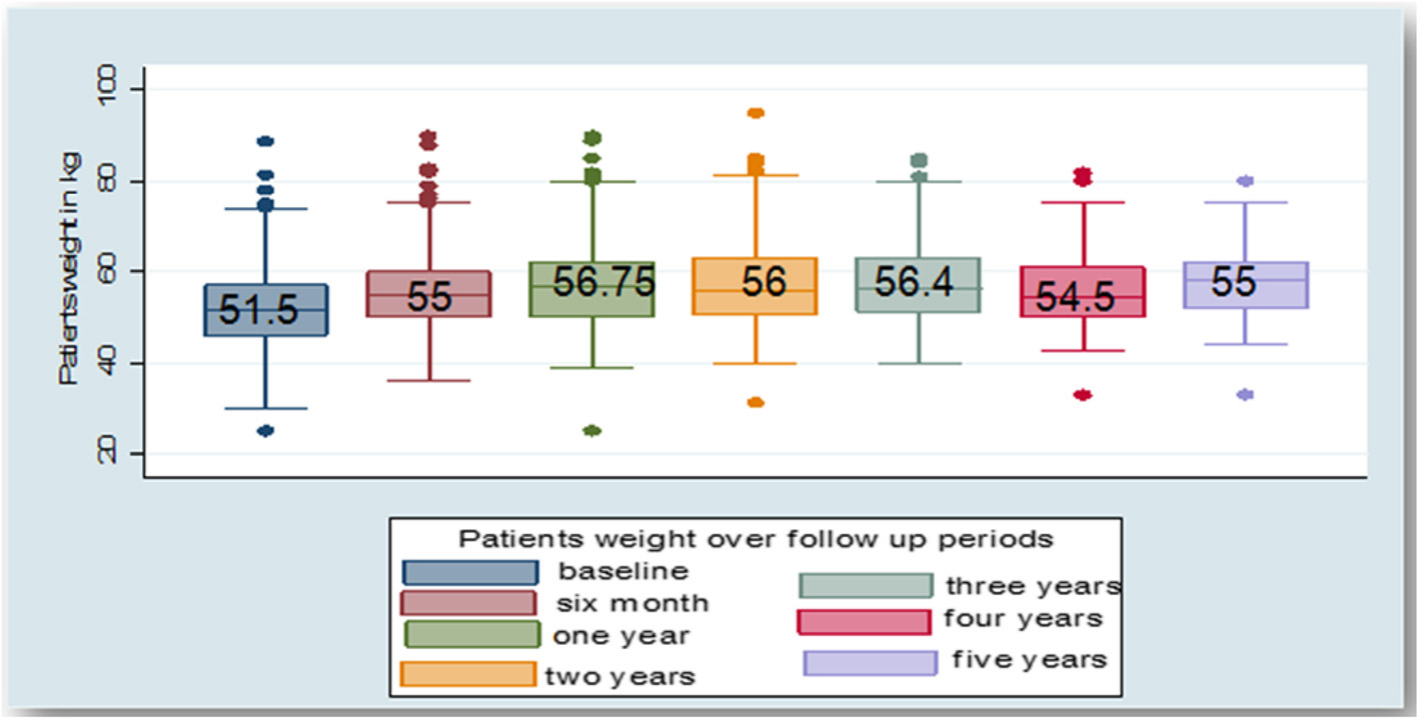
Progressive change in median weight over the follow up period in four selected health facilities of Kembata and Hadiya zones of SNNPR, Ethiopia [2007–2011]

**Fig. 2:**
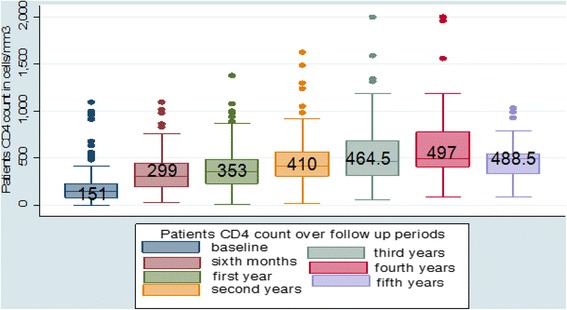
Progressive change in median CD4 Counts over the follow up period in four selected health facilities of Kembata and Hadiya zones of SNNPR, Ethiopia [2007–2011]

Regarding the functional status of the patients, 495 (68 %) were working, 194 (26.6 %) ambulatory and 39(5.4 %) bedridden at the time of the start of ARV treatment. Moreover, 413 (57 %) were in WHO stage III, 148(20.4 %) in stage II, 101 (14 %) patients were in stage I and 62 (8.6 %) patients were in stage IV at the time of ART initiation. Meanwhile, median [IQR] CD4 count was 151 [78, 224] cells/mm^3^. Six hundred seventy five (92.5 %) patients had good, 18(2.4 %) fair and 37(5.1 %) poor adherence to recommended ART regimen (Table 2). Median weight [IQR] of patients at baseline was 51.5 [46 57.2] kg and had increased to 55 kg and 56.75 kg(*p* = 001) at first 6 and 12 months follow up, respectively (Fig. 1). Similarly, CD4 count significantly increased over the last four consecutive years of follow up. The median CD4-cell count at ART initiation was 151 cells/mm^3^ which increased to 299 cells/mm^3^ at 6 months, 353 cells/mm^3^ at 12 months, 410 cells/mm^3^ at 24 months, 464.5 cells/mm^3^ at 36 months, 497 cells/mm^3^ at 48 months and 488.5 cells/mm^3^ at 60 months of follow up (Fig. 2).

**Table 2:** Clinical characteristics of patients in four selected health facilities of Kembata and Hadiya zones, 2007–2011 (*n* = 730)

Variables		Number	Percents
Opportunistic illness before initiation of ART	Had above one opportunistic infections	201	27.5 %
	Had one opportunistic infection	280	38.4 %
	No opportunistic Infection	249	34.1 %
Opportunistic illness after initiation of ART	Had above one opportunistic infections	26	3.5
	Had one opportunistic infection	170	23.3
	No opportunistic infection	534	73.2
CPT prophylaxis	Present	461	63.2 %
	Absent	269	36.8 %
Functional status	Working	495	68 %
	Bedridden	39	5.4 %
	Ambulatory	194	26.6 %
	Unknown	2	0.003 %
WHO clinical stage	Stage I	101	14.0 %
	Stage II	148	20.4 %
	Stage III	413	57.0 %
	Stage IV	62	8.6 %
	Unknown	2	0.003 %
Hemoglobin (g/dl)	<10	110	15.1 %
	≥10	620	84.9 %
CD4 counts at baseline	≤50 cells/mm^3^	106	14.5 %
	51 - 200 cells/mm^3^	398	54.5 %
	201 - 350 cell/mm^3^	195	26.7 %
	>350 cell/mm^3^	31	4.2 %
Weight at baseline	≤45	160	21.9 %
	46 – 55	329	45.1 %
	56 – 65	184	25.2 %
	>65	57	7.8 %
Regimen at baseline	1a = d4t-3TC-NVP	268	37.0 %
	1b = d4t-3TC-EFV	67	9.3 %
	1c = AZT-3TC-NVP	197	27.2 %
	1d = AZT-3TC-EFV	51	7.0 %
	TDF-3TC_NVP	141	19.5 %
	Unknown	6	0.008 %
Regimen adherence	Good	675	92.5
	Fair	18	2.4
	Poor	37	5.1
Treatment outcome	On treatment	423	57.9 %
	Dead	92	12.6 %
	Stopped	4	.5 %
	Lost	17	2.3 %
	Transfer out	109	14.9 %
	Drop	85	11.6 %

### Descriptive survival analysis of patients during the follow up period

**Fig. 3:**
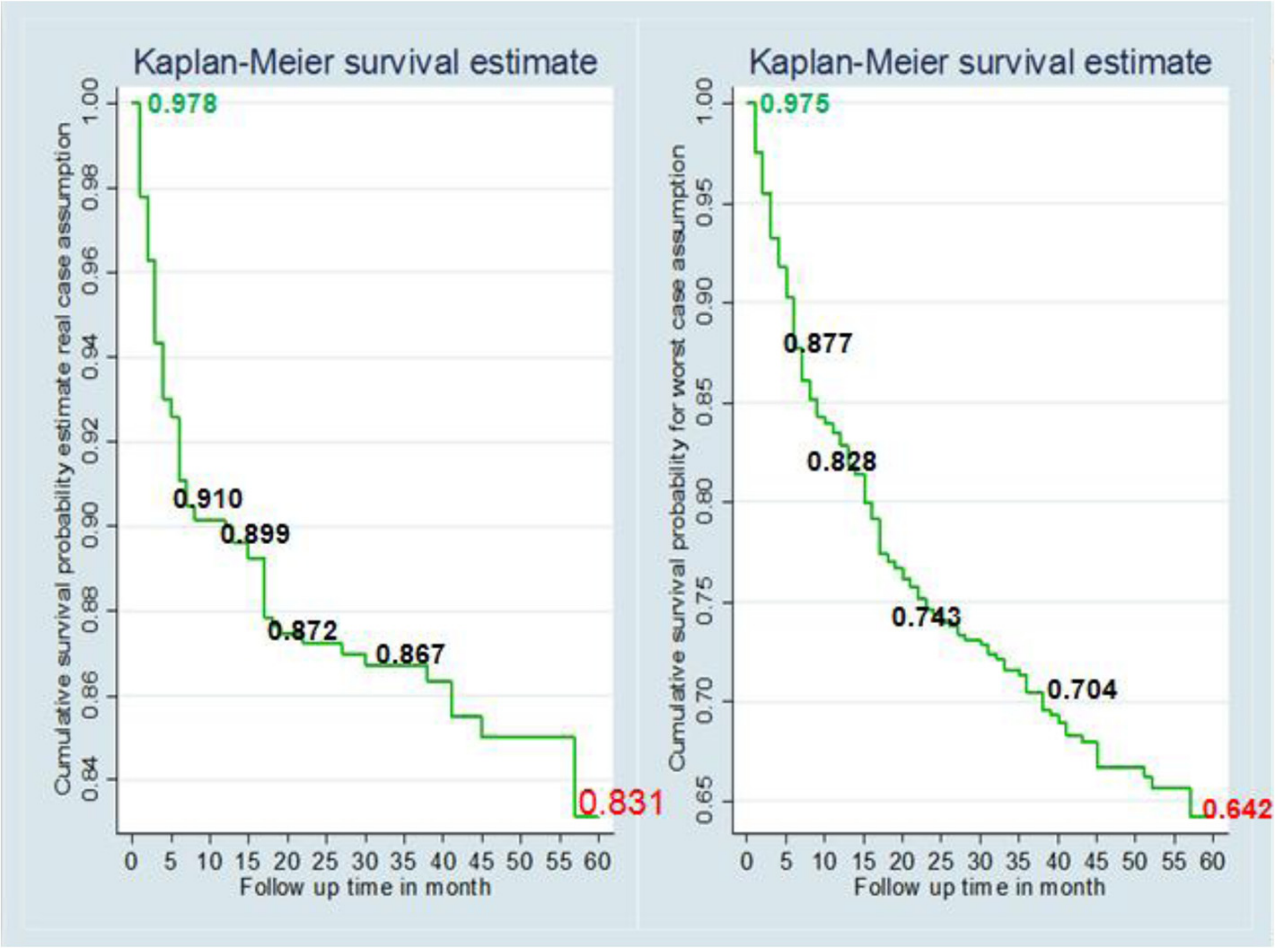
Cumulative survivals for real (death) and worst case (lost) assumptions of patients during follow up periods in four selected health facilities of Kembata and Hadiya zones of SNNPR, Ethiopia, 2007–2011

**Fig. 4:**
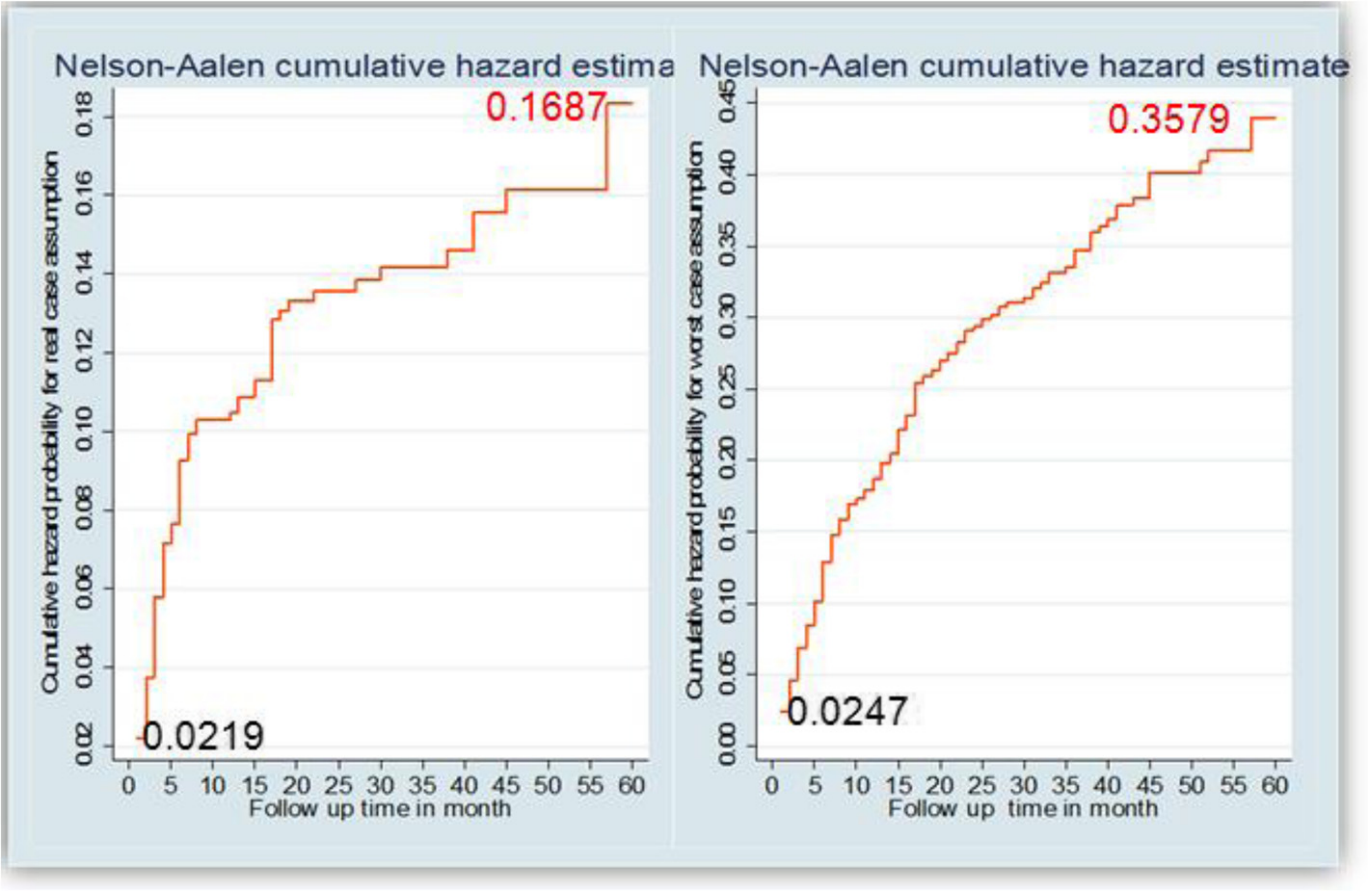
Cumulative Hazard function for real and worst case assumptions during follow up periods in four selected health facilities of Kembata and Hadiya zones of SNNPR, Ethiopia, 2007–2011

The median [IQR] follow-up time was 25 [9, 43] months. A total of 92(12.6 %) patients died in the five years follow up period after initiation of ARV treatment. Among these, 63 (68 %) deaths occurred in the first 6 months of treatment, 70 (76.1 %) deaths occurred within one year follow up, 14(15.2 %) between one and two years of follow up and the rest 8 (8.7 %) deaths occurred between third and fifth years of follow up. Overall, 106 (14.5 %) patients were lost to follow-up and 109(14.9 %) were transferred out to other heath facilities for treatment. The mortality rate during the follow-up period was 4/1000 person-years. Survival function showed that the probability of surviving for real and worst case assumptions was 0.83 and 0.64, respectively (Table 3). The survival function estimate graphs (Fig. 3) declined sharply at first six months follow up and then tailed off gradually. On the other side, the hazard estimate graphs (Fig. 4) sharply increased at the first six months of follow up. The initial sharp decline shows that there was high risk of dying during the first six months of follow up. The relatively long right tail was due to the few subjects who had long survival times. The minimum value of the survivorship function was not zero since the largest observed time was a censored observation.

**Table 3:** Kaplan Meier estimator of survival functions of event for both real and worst case assumptions of different follow up periods

Follow up time(month)	Events (death)	Survival function	95 % confidence interval	Events (worst cases)	Survival function	95 % confidence interval
1	16	0.98	[0.96, 0.99]	18	0.97	[0.96, 0.98]
3	25	0.94	[0.92, 0.96]	31	0.93	[0.91, 0.95]
6	22	0.91	[0.89, 0.93]	38	0.88	[0.85, 0.90]
12	7	0.90	[0.87, 0.92]	32	0.83	[0.80, 0.85]
24	14	0.87	[0.84, 0.89]	48	0.74	[0.71, 0.78]
36	2	0.87	[0.84, 0.89]	27	0.71	[0.67, 0.74]
48	4	0.85	[0.81, 0.88]	4	0.67	[0.63, 0.71]
60	2	0.83	[0.79, 0.87]	18	0.64	[0.59, 0.69]

**Fig. 5:**
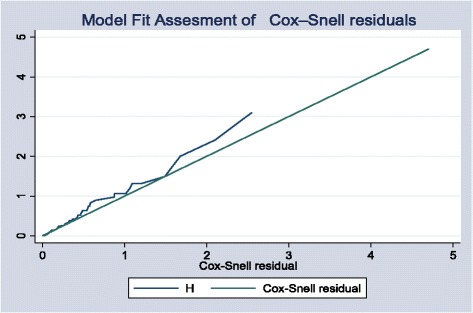
Overall model fit of the Cox regression using the Cox-Snell residuals

### Bivariate and multivariate analysis

Bivariate analysis of the factors revealed that the risk of death in five years follow up was associated with sex, spouse treatment status, tobacco smoking, other substance use, Tuberculosis (TB) status before initiation of ART, OIs after initiation of ART, TB after initiation of ART, functional status, WHO clinical stage, hemoglobin, baseline CD4 cells count, baseline weight, baseline drug regimen and regimen adherence. Factors that were associated with time to death at 25 % significant level in the bivariate analysis were included in the final multivariate analysis. According to the multivariate Cox model, the risk of death for patients with two or more opportunistic infections after initiation of ART was roughly three fold (hazard ratio, 3.353; 95 % CI, 1.462– 7.691) as compared with patients who had no opportunistic infection following the initiation of ART. Similarly, those with WHO stage III and IV at ART initiation had increased risk of death [hazard ratio (HR) 2.01, 95 % CI: 1.02,4.35; HR 2.42, (95 % CI: 1.19, 5.86, respectively], when compared with WHO stage I. However, those with 51–200 cells/mm^3^, 201–350 cell/mm^3^ and above 350 cell/mm^3^ baseline CD4 cell counts reduce the risk of death by 0.26 [95 % CI; 0.15, 0.45], 0.198 [95 % CI; 0.09, 0.43] and 0.12 [95 % CI; 0.02, 0.91], respectively as compared to patients with less than 51 cell/mm^3^ CD4 counts. Patients with hemoglobin level of 10 g/dl and above at initiation of ART had reduced a risk of death reduced by 0.22 [HR 0.22, 95 % CI: 0.14, 0.37] as compared to those whose hemoglobin level was less than 10 g/dl. The risk of death was 2.7 times higher for patients with poor regimen adherence (HR: 2.70, 95 % CI: [1.40, 5.20] as compared to patient who had good regimen adherence. Similarly, patients with ambulatory and bedridden functional status had higher risk of dying (HR: 1.79; 95 % CI: [1.05, 3.07]) and (HR: 3.40, 95 % CI: [1.61, 7.21]), respectively as compared with those working functional status at baseline (Table 4).

**Table 4:** Bivariate and Multivariate analysis for baseline clinical characteristics of patients in four selected health facilities of Kembata and Hadiya zones for Death (real case assumptions), 2007 – 2011

Characteristics real case	*N*	Bivariate analysis Hazard ratio7 (95 % CI)	Multivariate analysis Hazard ratio (95 % CI)
Sex			
Female	429	1	1
Male	301	1.69 (1.69, 2.12)	1.22 (0.70, 2.10)
Spouse treatment status			
Enrolled ART	179	1	1
Not Enrolled ART	254	2.54 (1.26, 5.13)	1.31 (0.29, 5.98)
Unknown	212	2.88 (1.41, 5.85)	1.32 (0.61, 2.86)
Smoke Tobacco			
No	636	1	1
Yes	94	1.70 (1.00, 2.87)	0.40 (0.12, 1.36)
Use other substances			
No	631	1	1
Yes	98	1.87 (1.13, 3.09)	1.12 (0.66, 1.54)
Attending HIV related education			
No	372	1	1
Yes	358	0.68 (0.44, 1.05)	0.87 (0.66, 1.90)
Understanding HIV prophylaxis			
Poor	374	1	1
Good	356	0.70(0.46, 1.06)	0.65 (0.45, 1.89)
Understanding of Adherence to HIV medication			
Poor	419	1	1
Good	311	0.68 (0.44, 1.05)	0.68 (0.38, 1.44)
OIs before initiation of ART			
No OIs	249	1	
Only one OIs	280	1.27 (0.78, 2.07)	NA
≥2 OIs	201	0.73 (0.44, 1.23)	NA
TB status before initiation of ART			
Absent	711	1	
Present	19	3.33 (1.54, 7.20)	1.25 (0.68, 1.54)
OIs after initiation of ART			
No OIs	534	1	1
Only one OIs	170	2.38 (1.51, 3.75)	1.24 (1.06, 1.89)
≥2OIs	26	10.90 (6.13, 19.37)	3.35 (1.46,7.69)
TB after initiation of ART			
Absent	662	1	1
Present	68	1.82 (1.03, 3.22)	1.428 (1.28, 3.39)
Past prophylaxis			
Not Received	269	1	
Received	461	0.88 (0.59, 1.33)	NA
Functional status			
Working	495	1	1
Ambulatory	194	2.81 (1.79, 4.41)	1.79 (1.05, 3.07)
Bedridden	39	6.32 (3.42, 11.700)	3.40 (1.61, 7.21)
WHO clinical stage			
Stage I	101	1	1
Stage II	148	2.32 (0.64, 8.41)	NA
Stage III	413	5.06 (1.59, 16.13)	2.01 (1.02, 4.35)
Stage IV	62	8.58 (2.46, 19.87)	2.42 (1.19, 5.86)
Hemoglobin by g/dl			
<10	110	1	1
≥10	620	0.17 (0.11, 0.26)	0.22 (0.14, 0.37)
CD4 counts at baseline			
<51 cells/mm^3^	106	1	1
51 –200 cells/mm^3^	398	0.23 (0.15, 0.35)	0.26 (0.15, 0.45)
201 –350 cell/mm^3^	195	0.13 (0.07, 0.25)	0.20 (0.09, 0.43)
>350 cell/mm^3^	31	0.06 (0.01, 0.46)	0.12 (0.02, 0.91)
Weight at baseline			
≤45	160	1	1
46 –55	329	0.58 (0.37, 0.93)	0.52 (0.29, 1.93)
56 – 65	184	0.39 (0.21, 0.70)	0.51 (0.28, 1.18)
>65	57	0.24 (0.07, 0.75)	0.55 (0.16, 1.19)
Regimen at baseline			
1a = d4t-3TC-NVP	268	1	1
1b = d4t-3TC-EFV	67	0.71 (0.32, 1.58)	NA
1c = AZT-3TC-NVP	197	0.55 (0.31, 0.97)	1.63 (0.87, 3.05)
1d = AZT-3TC-EFV	51	0.84 (0.36, 1.98)	NA
TDF-3TC_NVP	141	1.39 (0.82, 2.35)	NA
Regimen Adherence			
Good	675	1	1
Fair	18	4.36 (2.00, 9.50)	1.66 (0.59, 4.62)
Poor	37	5.94 (3.49, 10.14)	2.70 (1.40, 5.20)

*OIs* Opportunistic infection

*NA* Not available

## Discussion and conclusions

The proportion of female patients was significantly higher than the one of male patients. This might be due to the fact that women by their nature are highly vulnerable to HIV infection than their counterparts. Moreover, women may be more often diagnosed than men due to high frequency of testing for women as part of PMTCT program. Similar findings were reported from Hawassa referral hospital in SNNPR, Ethiopia [13]. Moreover, the findings of this study coincide with other studies conducted in Africa in terms of sex proportionality [14, 15].

About 12.2 % of patients died during the five years follow up period and 68 % of the deaths occurred within the first six months of follow up. This was higher than 11.2 % death after 24 months of follow up reported in the retrospective longitudinal study from Ethiopia [16]. The overall death in the five year follow up study was lower compared to 29.7 % death after three years of follow up from Cameroon [17] and 28.5 % from Tanzania [18] who died after five years of ART follow up. However, the total death in our finding was slightly higher than the two years follow up study conducted in Shashamene and Assela Hospitals [10.2 %] from Ethiopia [19]. This could be explained by the fact that our study was based on five years of survival compared to the two years of survival analysis [19].

The higher mortality in the first few months of therapy in our set up was similar to other studies reported from different African countries [17–20]. This may partly be explained by the fact that most of the patients had CD4 count less than 200 cells/mm^3^ at the time of ART initiation and were at an advanced WHO stages [III and IV]. More than half of the patients did not attend any kind of HIV related education and had no idea about prophylaxis. Besides, 48.6 % of them were from rural areas, where access to information and Volunteer counseling and testing services were highly limited during initiation of ART. These might have been the factors responsible for delayed diagnosis which in turn has led to high early mortality. Other study results from South Africa showed that one or more opportunistic diseases and immune reconstitution inflammatory syndrome were the major causes of death at early initiation of ART [21].

Lost to follow up was found to be high, 106(14.5 %). Most of the lost to follow up cases occurred within six months of starting ART. This was lower than the national dropout estimate of 21.3 % from Ethiopia [22]. Moreover, lost to follow up was higher when compared to other studies in Africa. Studies from Cameroon and Tanzania reported that 5 and 9.7 % lost in the two years follow up periods, respectively [17, 18]. The longer follow up period is believed to be the reason for the high lost to follow up rate in our study. The addictive behavior of the patients, drug side effect, absence of tangible information on the enrolment of spouses and long distance travel to take medicines might have discouraged the patients to miss follow ups. Moreover, the lost cases in our context had probably been dead but were not reported due to poor patient tracing and reporting mechanisms of the recent scaled up services of the study facilities. The lack of National death registration has limited the capacity of tracing lost cases for possible confirmation of outcome [22].

WHO clinical stage of disease was a strong predictor of mortality in this study. Patients with advanced WHO clinical stages III and IV had nearly twice higher risk of dying during the five years survival as compared with reference group of WHO clinical stage I. Several studies from Africa including Ethiopia have shown that advanced WHO stage III and IV was a strong predictor of mortality in patients on ART, even after controlling for CD4 cell count [19, 23–26]. The possible reason for the higher risk of death in patients with advanced clinical disease stages III and IV could be the increased susceptibility for contracting opportunistic infections among this group.

The five years survival analyses result showed that patients who started ART with lower CD4 cells count had an increased risk of death compared to those who started with higher CD4 cell counts. The improved rate of survival among patients who initiated antiretroviral therapy at higher CD4 cells count probably can be attributed to multiple factors, including earlier control of viral replication and viral diversity and a greater immunologic benefit [27]. These findings are in line with numerous study findings from resource-limited settings showing low baseline CD4 cell count was a major predictor of death, and waiting ART until the CD4 cell count falling below 350 cells/mm^3^ was associated with increased morbidity and mortality [28, 29]. Similarly, findings from the developed world suggested that starting ART at higher CD4 cell counts improved treatment outcomes and decreases mortality [25, 30]. Moreover, recent three years survival analysis result from Hawassa referral Hospital of SNNPR, Ethiopia has showed that patients with CD_4_ count below 50 cells/mm^3^ had higher risk of death [10].

Hemoglobin values at baseline were strongly associated with the risk of death. Similar study findings were reported from Tanzania [18] and Ethiopia [13, 19]. Anemia could be an indicator of advanced disease or clinical feature of some opportunistic infections which might aggravate risk of death. Furthermore, anemia can be a feature of certain opportunistic diseases, like disseminated mycobacterial infection and parvovirus B19 [31]. Several other etiologic factors may be involved in the development of HIV-associated anemia, including micronutrient deficiencies, immunological myelosuppression, impaired erythropoietin production and blood loss from intestinal opportunistic disease [31].

Baseline functional status was strongly associated with five years survival time. The risk of death for those who started ART at baseline with bedridden functional status was twice higher than those who started ART with working functional status. Similarly, recent study finding from Hawassa referral Hospital, SNNPR Ethiopia has showed that patients with bedridden functional status had higher risk of death as compared to those with working functional status [13]. However, there is limited data from Africa trying to explain higher mortality rates among the patients with functional status. The higher risk of death among patients with bedridden functional status could be the increased susceptibility for opportunistic infections and other clinical complications including bedsore, thrombosis, malnutrition, chronic illness and anorexia.

Patients with poor regimen adherence had increased risk of death [HR 2.695, 95 % CI: 1.396–5.203] as compared with those who had good adherence of regimen. Medication adherence is critically important to achieve viral suppression and to avoid the emergence of viral resistance. The presence of more than one opportunistic infection after ART initiation had also an effect on the five year survival. Those patients diagnosed as having two or more opportunistic infections after ART initiation, had a much greater likelihood of dying than those who had no or only one opportunistic infection. Similarly, numerous study results from different settings have revealed that opportunistic infection after ART initiation had resulted in increased risk of death [16, 22, 32, 33]. The fact that patients develop opportunistic infections after ART is an indication of treatment failure and poor immunological response of ART which might lead to higher mortality. Moreover, in this study opportunistic infection after ART could be immune reconstitution inflammatory syndrome which has been shown to be one of the causes of mortality in HIV/AIDS patients on ART [21]. Pulmonary TB infection after ART initiation has statistically significant association with the risk of death. A study from three specialized hospitals of Oromia region, Ethiopia, revealed that TB/HIV co-infected patients had a lower quality of life as compared with those HIV infected patients without TB infection [34].

Our study findings revealed that CD4 count and weight were significantly increased after initiation of ART at five years survival time. Similarly, numerous study results from different settings revealed that CD4 count and weight of patient were increased after initiation of ART [14, 15, 17].

This five years survival analysis, in both models, sex, age, body weight, past opportunistic infection, place of residence, religious affiliation, facilities of care and level of education, marital status, risk behaviors such smoking tobacco and use of other substances were not statistically significant in multivariate analysis. Similar results were reported in different studies conducted in developing countries (37, 40, 46_47).

The collection of data from large number of patients, inclusion of ART services at different health facility levels (hospitals and health centers) as opposed to the hospital studies from Ethiopia [13, 19, 32], inclusion of all eligible patient cards from the study facilities and use of individual level data were some of the strengths of this study. However, the study has limitations as well, since secondary data was used for statistical analyses, exploring a wide range of possible predictors for treatment outcomes was not possible. Thus, it was not possible to measure some important predictors such as nutritional status, viral loads and other important variables established as predictors of morbidity and mortality in patients on ART [21]. Retrospective nature of the study possibly introduces bias as the quality of information obtained from these finding is highly dependent on the completeness of patient records. Moreover, these findings were limited to Ethiopia and the first five years of ART.

## Conclusion

 The high early mortality have practical implications for addressing the need to initiate ART early which would require early diagnosis of HIV and might be addressed with improved counseling and testing services at facilities with strong community education and mobilization. The various baseline factors including WHO staging, CD4 counts, functional status, regimen adherence and hemoglobin level have significant effect on five years survival. Substantial efforts need to be made to move patients into care earlier in their disease progression in order to obtain the maximum benefit from ART. CD4 counts and weight of the patients were significantly increased at different follow up period. Health care providers should give serious attention to the identification of HIV opportunistic infections during initiation of ART which could contribute to reducing early death. In conclusion, all people living with HIV/AIDS should initiate ART as early as possible. Initiation of ART at the early stages of the disease and before the deterioration of the functional status of the patients and before the reduction of CD4 counts and hemoglobin levels with an intensified health education on adherence to ART regimen is recommended.

## Abbreviations

AIDS: Acquired Immune Deficiency Syndrome
ART: Anti retroviral treatment
HIV: Human Immunodeficiency Virus
HR: Hazard ratio
IQR: Inter quartile range
SNNPR: South Nation Nationalities and Peoples Region
TB: Tuberculosis
